# Identification of a Fragment-Based Scaffold that Inhibits the Glycosyltransferase WaaG from *Escherichia coli* †

**DOI:** 10.3390/antibiotics5010010

**Published:** 2016-01-28

**Authors:** Claudio Muheim, Amin Bakali, Olof Engström, Åke Wieslander, Daniel O. Daley, Göran Widmalm

**Affiliations:** 1 Arrhenius Laboratory, Department of Biochemistry and Biophysics, Stockholm University, Stockholm S-106 91, Sweden; claudio.muheim@dbb.su.se (C.M.); aminbak@gmail.com (A.B.); 2 Arrhenius Laboratory, Department of Organic Chemistry, Stockholm University, Stockholm S-106 91, Sweden; j.olof.engstrom@gmail.com

**Keywords:** glucosyltransferase, Gram-negative bacteria, lipopolysaccharide, fragment-based lead discovery, scaffold

## Abstract

WaaG is a glycosyltransferase that is involved in the biosynthesis of lipopolysaccharide in Gram-negative bacteria. Inhibitors of WaaG are highly sought after as they could be used to inhibit the biosynthesis of the core region of lipopolysaccharide, which would improve the uptake of antibiotics. Herein, we establish an activity assay for WaaG using ^14^C-labeled UDP-glucose and LPS purified from a ∆*waaG* strain of *Escherichia coli*. We noted that addition of the lipids phosphatidylglycerol (PG) and cardiolipin (CL), as well as the detergent 3-[(3-cholamidopropyl)dimethylammonio]-1-propanesulfonate (CHAPS) increased activity. We then use the assay to determine if three molecular scaffolds, which bind to WaaG, could inhibit its activity *in vitro*. We show that 4-(2-amino-1,3-thiazol-4-yl)phenol inhibits WaaG (IC_50_ 1.0 mM), but that the other scaffolds do not. This study represents an important step towards an inhibitor of WaaG by fragment-based lead discovery.

## 1. Introduction

The outer membrane (OM) of Gram-negative bacteria is an asymmetric bilayer, composed of phospholipids in the inner leaflet and lipopolysaccharide (LPS) in the outer leaflet. LPS is structurally divided into three parts: a hydrophobic lipid A that anchors the LPS molecule to the bacterial outer membrane, a phosphorylated core oligosaccharide (OS) that comprises ~10 sugar residues [1] and a repeating oligosaccharide unit referred to as the *O*-antigen polysaccharide [2]. The core OS can be subdivided into a conserved inner core that contains 3-deoxy-d-*manno*-oct-2-ulosonic acid (Kdo) and l-*glycero*-d-*manno*-heptose (Hep) and a less conserved outer core that consists of hexose residues.

The LPS layer acts as a permeability barrier for small molecules; thus, essential nutrients and ions can only access the cell through β-barrel porins that are embedded in the outer membrane [3]. Porins allow passive diffusion of soluble molecules with a molecular mass of <600 Da, which restricts the passage of many antibiotics (known as intrinsic resistance). However, when LPS biosynthesis is inhibited by gene knockout, the LPS is truncated, and cells are more permeable to antibiotics [4]. Inhibition of LPS biosynthesis has therefore been highlighted as a promising strategy for combatting intrinsic resistance to antibiotics [5], and a number of groups have looked for inhibitors [6,7,8,9,10].

**Figure 1:**
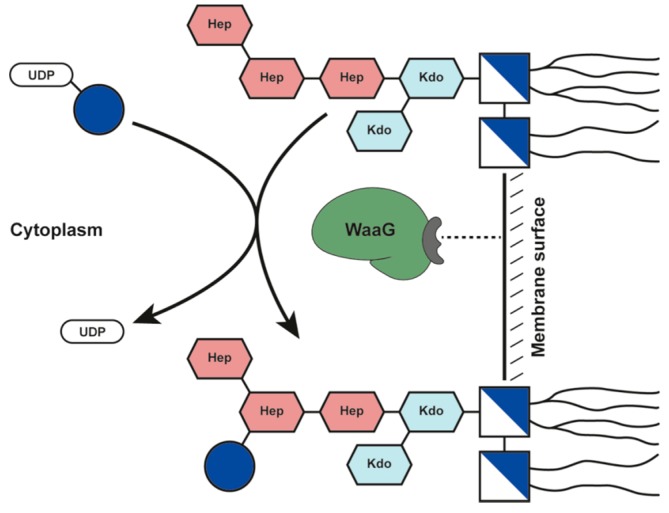
Schematic of the action of the glycosyltransferase WaaG transferring a d-glucose residue (blue ball) from UDP-glucose to heptose-II of the inner core of an LPS molecule.

One promising target for the development of inhibitors is the α-1,3-glucosyltransferase I (WaaG), which transfers glucose from UDP-glucose to the l-*glycero*-d-*manno*-heptose-II (HepII) residue of the inner core (Figure 1). The deletion of the gene encoding WaaG therefore results in an inability to synthesize the outer core OS and the *O*-antigen polysaccharide (termed a deep rough phenotype) [11]. The resulting LPS layer also has an 80% reduction in heptose phosphorylation of the inner core OS, which is detrimental for the OM stability [12]. In *Salmonella enterica*, deletion of the gene encoding WaaG abrogates the ability to enter epithelial cells [13]. Most importantly, deletion of the gene encoding WaaG causes increased susceptibility to seven classes of antibiotics [4] and therefore indicates that WaaG is a valid target for antibiotic potentiators. These potentiators could be used to inhibit LPS biosynthesis in pathogenic and multi-drug-resistant strains of Gram-negative bacteria, thus making them susceptible to classes of antibiotics that are normally reserved for Gram-positive bacteria.

Recently we initiated a fragment-based lead discovery (FBLD) approach to identify an inhibitor of WaaG. FBLD has become increasingly popular in recent years, as it captures more chemical diversity space than a conventional high-throughput screen [14,15]. It involves the screening of low molecular weight molecules (typically <250 Da) for low affinity binders (*K*_D_’s from high µM to low mM) and then elaborating or linking these binders to produce a high-affinity inhibitor [14,16]. Our initial screen of the Maybridge fragment-based library by NMR spectroscopy identified three different molecular scaffolds that bind to WaaG and at the same time compete with UDP [17]. These molecules were by nature weak binders with *K*_D_’s ranging from 0.5 to 1 mM. Importantly, they all obeyed the “rule of three” [18], which is a set of rules that have been formulated to determine which molecules are most likely to be good leads. In the present study, we have established a biochemical assay for WaaG and used this assay to evaluate the potency of these molecular scaffolds. Our data indicate that one of them, 4-(2-amino-1,3-thiazol-4-yl)phenol, can weakly inhibit WaaG *in vitro* and may therefore serve as a lead molecule for the development of an inhibitor.

## 2. Results and Discussion

### 2.1. An in Vitro Assay for WaaG

**Figure 2:**
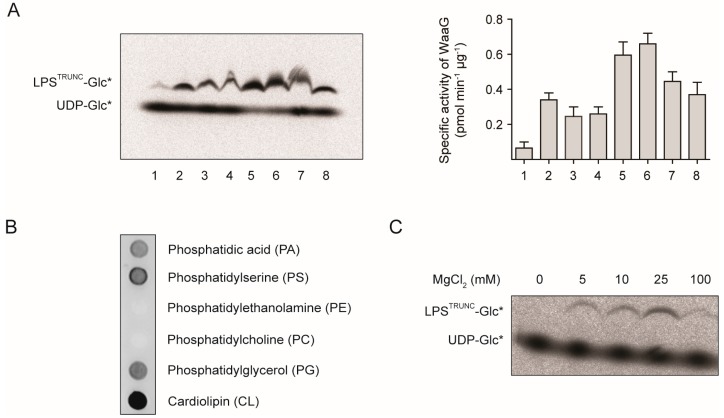
His-WaaG activity under different lipid compositions. (**A**) Mixed micelles containing CHAPS and/or various lipids were incubated with LPS^TRUNC^ (0.5 µg·mL^−1^), UDP-Glc* and His-WaaG (0.1 mg·mL^−1^) to monitor LPS-glycosylation. After 1 h, the reaction was stopped by the addition of Laemmli buffer, and the products were separated by SDS-PAGE and detected by digital autoradiography. Lane 1: no added lipids or detergents; Lane 2: 20 mM CHAPS; Lane 3: 10 mM PG; Lane 4: 5 mM CL; Lane 5: 20 mM CHAPS + 10 mM PG; Lane 6: 20 mM CHAPS + 10 mM PG + 1 mM CL; Lane 7: 20 mM CHAPS + 10 mM PG + 5 mM CL; Lane 8: 3% DHPC. Quantification of LPS^TRUNC^-Glc* and UDP-Glc* was performed with ImageJ and the specific activity calculated (see the Experimental Section). Specific activities plotted as the mean ± SD; *n* = 2. (**B**) Nitrocellulose membranes containing immobilized lipids (100 pmol/spot) were incubated with purified His-WaaG (3 µg·mL^−1^). After washing to remove unbound protein, His-WaaG that bound to different lipids was detected by immunoblotting with antisera to the His-tag. (**C**) His-WaaG activity was monitored *in vitro* with different concentrations of MgCl_2_.

Our first aim was to establish an *in vitro* activity assay for WaaG. Initially, we mixed purified His-WaaG with ^14^C-labeled UDP-glucose (UDP-Glc*) and truncated LPS (LPS^TRUNC^) that had been purified from a ∆*waaG* strain of *E. coli.* Thus, the ^14^C-labeled glucose (Glc*) could be enzymatically transferred from UDP-Glc* to the HepII residue of the inner core of the LPS^TRUNC^. Following incubation for one hour at room temperature and analysis by SDS-PAGE, we observed the formation of a weak band corresponding in molecular mass to LPS^TRUNC^-Glc* (Figure 2A, Lane 1). Importantly, the formation of this band was not observed when we used LPS that had been purified from a wild-type strain of *E. coli* or when His-WaaG or LPS^TRUNC^ were omitted. These data indicate that His-WaaG is able to transfer the Glc* from UDP-Glc* to LPS^TRUNC^.

Since WaaG binds peripherally to the inner membrane [19,20], we investigated if different lipids would influence its activity *in vitro*. We incubated a nitrocellulose membrane that had been spotted with immobilized lipids with His-WaaG. The nitrocellulose membrane was then washed, and the detection of His-WaaG was carried out with antisera raised to the His-tag. His-WaaG bound efficiently to four out of six lipids on the nitrocellulose membrane (Figure 2B). Two of these lipids, phosphatidylglycerol (PG) and cardiolipin (CL), are present in *E. coli* membranes [21]. Notably, His-WaaG did not bind to phosphatidylethanolamine (PE), which is the major lipid in *E. coli*. We also observed binding of His-WaaG to phosphatidic acid (PA) and phosphatidylserine (PS), which are precursors in phospholipid synthesis and only minor components of *E. coli* membranes. Taken together, these findings suggest that the positively-charged surface of His-WaaG [22] might interact with negatively-charged phospholipids (PA, PS, PG and CL), but not with zwitterionic phospholipids (PE, PC).

Since His-WaaG binds to PG and CL, we repeated the *in vitro* assay in the presence of different combinations and concentrations of these lipids. We also included CHAPS, a zwitterionic detergent that controls micelle size and shape. Furthermore, we tested DHPC, a short-chain phosphatidic acid derivative that has been used to solubilize rough LPS molecules [23]. All of the conditions tested improved the activity of His-WaaG (Figure 2A, Lanes 2 to 8). Although we did not explore the lipid concentrations and combinations exhaustively, we were able to obtain a set of conditions that were optimal for our studies. These conditions included either 20 mM CHAPS, 10 mM PG or 20 mM CHAPS, 10 mM PG and 1 mM CL (Figure 2A, Lanes 5 and 6). It is also worth noting that LPS-OH, where ester-linked acyl chains are removed by aqueous ammonia, was also effective as an acceptor for the radioactive glucose. Thus, lipid A devoid of some or all of the ester-linked lipids attached to the glucosamine residues does not impact the activity.

During the course of this work, Qian *et al.* developed a slightly different *in vitro* assay to study the assembly of the outer core of LPS from *E. coli* K-12 [24]. They produced a ∆*waaG* LPS variant consisting of heptose_2_-1-dephospho Kdo_2_-lipid A, which subsequently acted as an acceptor for the transfer of d-glucose from UDP-glucose by the action of WaaG. The monophosphorylated LPS derivative facilitated the separation and analysis on silica-based TLC plates using a four-component solvent system. In addition to being able to follow the reaction by using ^14^C-labeled glucose, they also utilized ^32^P-Hep_2_-1-dephospho Kdo_2_-lipid A as a substrate, which made it possible to differentiate the acceptor from products formed through the action of one or more glycosyltransferases. In the study, they also noted that WaaG hydrolyzes UDP-glucose in the absence of the LPS acceptor, a finding that we previously also observed [17]. However, Qian *et al.* observed the highest specific activity in the absence of MgCl_2_, when Hep_2_-1-dephospho Kdo_2_-lipid A was used as an acceptor. In contrast, we observed no activity in the absence of MgCl_2_. In our *in vitro* assay, the highest activity was observed with 25 mM MgCl_2_ (Figure 2C).

### 2.2. L1 Can Inhibit WaaG in Vitro

**Figure 3:**
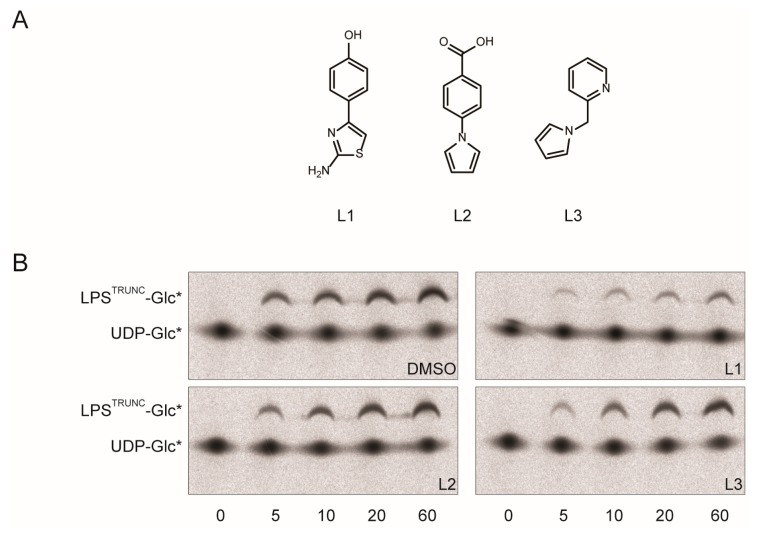

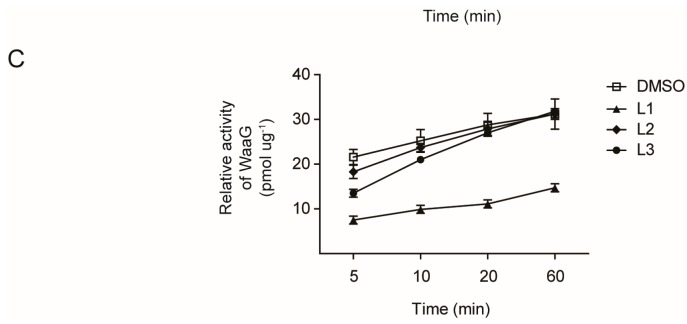
Compound L1 inhibits His-WaaG. (**A**) Structures of ligands: L1, 4-(2-amino-1,3-thiazol-4-yl)phenol; L2, 4-(1H-pyrrol-1-yl)benzoic acid; L3, 2-(1H-pyrrol-1-ylmethyl)pyridine. (**B**) Mixed micelles containing 20 mM CHAPS, 10 mM PG and 1 mM CL were mixed with LPS^TRUNC^ (0.5 µg·mL^−1^) and UDP-Glc*. The reaction was started by adding His-WaaG (0.1 mg·mL^−1^) and incubated either with 2.5% DMSO (*v*/*v*) or 25 mM of compound L1, L2 or L3. Samples were collected after different time points, inactivated by adding Laemmli buffer, separated by SDS-PAGE and detected by autoradiography. (**C**) Quantification of the activity of His-WaaG in the presence of either DMSO or 25 mM L1, L2 or L3. Gels in (B) were quantified by densitometry, and the activity was calculated as described in the Experimental Section.

Previously, we identified three small molecular scaffolds that bind to WaaG *in vitro* (Figure 3A) [17]. To determine if these molecular scaffolds were suitable for the development of an inhibitor, we added 25 mM of them to the *in vitro* assay and monitored the formation of LPS^TRUNC^-Glc* over time. When compound L1 (4-(2-amino-1,3-thiazol-4-yl)phenol) was added, we noted that it drastically reduced the amount of LPS-Glc* formed (Figure 3B, upper right panel; Figure 3C). In contrast, the fragment-based molecules L2 and L3 had no significant effect on the formation of LPS^TRUNC^-Glc* (Figure 3B, lower panels; Figure 3C).

**Figure 4:**
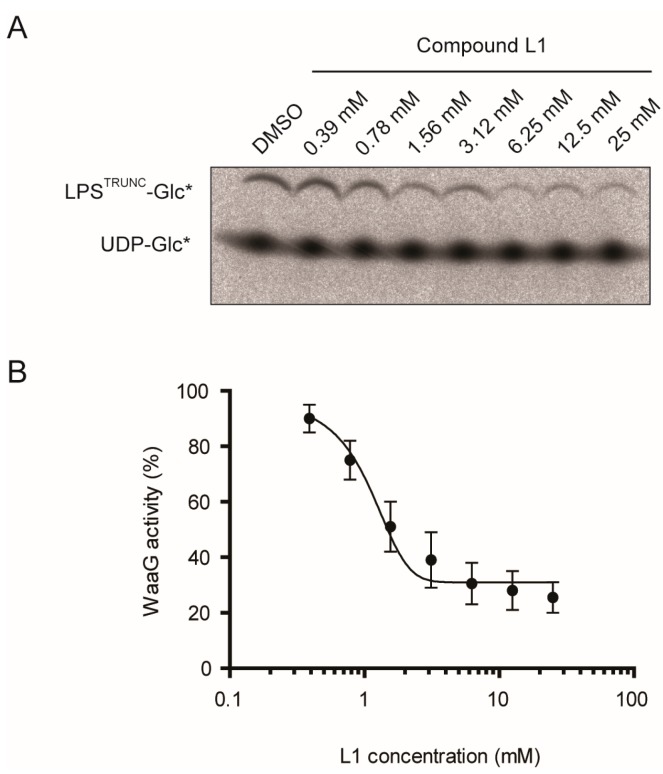
Dose-response curve for compound L1. (**A**) To evaluate the IC_50_, the activity of His-WaaG was assayed in the presence of 20 mM CHAPS, 10 mM PG, 1 mM CL and different concentrations of L1. Samples were collected after 1 h and inactivated by adding Laemmli buffer. The products were separated by SDS-PAGE, detected by digital autoradiography and quantified by densitometry. The IC_50_ was calculated as the midpoint between the DMSO control (which was assumed to be 100% activity) and the activity at 25 mM of L1 (which was assumed to be 0% activity). (**B**) His-WaaG activity is shown as a function of L1 concentration. The resulting IC_50_ value for compound L1 was calculated to be 1.0 mM (*n* = 2).

To better understand how potent L1 was, we monitored the formation of LPS^TRUNC^-Glc* in the presence of various concentrations of L1 (Figure 4A). We then used these data to plot a dose-response curve, and we calculated the IC_50_ to be 1.0 mM (Figure 4B). Although this level of inhibition is weak, it is comparable to initial hits against other targets in fragment-based screens. Many of these hits have been evolved into potent inhibitors [16].

## 3. Experimental Section

### 3.1. Bacterial Strains, Plasmids and Chemicals

The plasmid pET-M11 (EMBL, Heidelberg, Germany), which encodes for an N-terminal His_6_-tag followed by a linker (PMSDYDIPTTENLYFQGA) and WaaG [17], was used to overexpress His-WaaG in *E. coli* BL21-AI (Invitrogen, Stockholm, Sweden). The *E. coli* strain CWG303 derived from F470 with a *waaG*::*aacC1* insertion [11] was used to extract LPS. Antibiotics and compounds L1 to L3 were obtained from Sigma-Aldrich (Stockholm, Sweden). DHPC was purchased from Avanti Polar Lipids (Alabaster, AL, USA).

### 3.2. Protein Expression and Purification of E. coli His-WaaG

The vector pET-M11 encoding for His-WaaG was transformed by heat shock into BL21-AI cells (Invitrogen) and plated on LB agar plates containing 50 μg·mL^−1^ kanamycin and 7.5 μg·mL^−1^ tetracycline. A single colony was inoculated into 20 mL LB medium containing 50 μg·mL^−1^ kanamycin and incubated overnight at 37 °C and 200 rpm. The culture was then diluted into 1 L of LB medium containing 50 μg·mL^−1^ kanamycin, and incubation was continued until an OD_600_ of ~0.5 was reached. The temperature was then lowered to 22 °C, and protein expression was induced at an OD_600_ of ~0.6 with 0.5 mM IPTG and 0.2% (*w*/*v*) l-arabinose; growth was carried out for 20 h. Cells were subsequently harvested by centrifugation (8000× *g* for 20 min), and purification was performed as described previously [22] with some modifications. Cells were resuspended in 140 mL of Buffer A (20 mM Tris-HCl, pH 8.0, 300 mM NaCl, 0.5% Triton X-100, 1 mM TCEP) and then lysed on ice by sonication for 10 min (VibraCell ultrasonic processor; 10-s pulses at 40% amplitude were interleaved with 10-s delays without irradiation). The lysate was centrifuged (10,000× *g* for 30 min), and the supernatant was incubated with 5 mL Ni-NTA Agarose resin (Qiagen, Stockholm, Sweden). After washing twice with 50 mL of Buffer B (Buffer A without Triton X-100) containing 20 mM imidazole, His-WaaG was eluted with 30 mL of Buffer B containing 250 mM imidazole. The eluate was concentrated by using a Vivaspin 20 microconcentrator (30-kDa cut-off; Sartorius AG, Göttingen, Germany) and purified with a HiPrep 16/60 Sephacryl™ S-200 HR column (GE Healthcare, Uppsala, Sweden). The purified protein was then concentrated to 5 mg·mL^−1^, and aliquots were stored at −20 °C.

### 3.3. LPS Extraction

A single colony of *E. coli* CWG303 was inoculated into 20 mL LB medium containing 15 μg·mL^−1^ gentamycin and incubated for 8 h at 37 °C and 200 rpm. The culture was then diluted into 1 L of LB medium containing 15 μg·mL^−1^ gentamycin, and incubation was continued overnight. Cells were harvested by centrifugation (8000× *g* for 20 min), and the pellet was washed successively with deionized water, 95% ethanol, acetone (twice) and diethyl ether (50 mL each). Dried cells of a culture volume of 4 L were pooled (~3.4 g), and LPS was extracted by using the phenol/chloroform/petroleum ether (PCP) extraction method [25,26]. The LPS yield was in the range of 60 to 80 mg.

### 3.4. O-Deacylation of LPS in Aqueous NH_4_OH

PCP-extracted LPS (40 mg) from *E. coli* CWG303 was solubilized in 6 mL aqueous 12.5% NH_4_OH [27], and the mixture was stirred overnight at room temperature. The reaction was neutralized to pH 7 with 37% HCl (aq), and polyvalent metal ions were removed by Chelex-100 treatment. The *O*-deacylated LPS (LPS-OH) was dialyzed for 8 h (2-kDa cut-off, Spectrum labs, Stockholm, Sweden) four times against 5 L of deionized water to remove excessive salts. The sample was freeze-dried (Lyo Chamber Guard, Christ LCG, Osterode am Harz, Germany) to yield ~20 mg of the LPS-OH.

### 3.5. In Vitro Lipid Binding Assay

To assess the binding of His-WaaG to different lipids, a nitrocellulose membrane containing immobilized lipids (P-6002, Echelon, Salt Lake City, UT, USA) was incubated with purified His-WaaG according to the instructions of the manufacturer. The membrane was blocked with 1% (*w*/*v*) dry milk in 10 mM sodium phosphate pH 7.4 and 150 mM NaCl for 1 h at room temperature to prevent non-specific binding of the antibody, followed by incubation with purified His-WaaG (3 µg·mL^−1^) for 1 h at room temperature. The membrane was washed (3 × 15 min) in wash buffer (10 mM sodium phosphate pH 7.4, 150 mM NaCl) and then incubated with anti-His antibody (monoclonal 1:2000, Qiagen) for 1 h at room temperature. Subsequently, the membrane was washed three times in wash buffer and incubated with goat anti-mouse HRP antibody (1:5000 dilution, Novagen, Solna, Sweden) for 1 h at room temperature. Finally, the membrane was washed again three times in wash buffer, and chemiluminescent detection was performed using ECL detection solutions (ECL detection kit, GE healthcare) according to the instructions of the manufacturer.

### 3.6. WaaG Activity Assay

LPS extracted from *E. coli* CWG303 was mixed with CHAPS (Sigma), 1,2-dioleoyl-*sn*-glycero-3-[phospho-*rac*-(3-lysyl(1-glycerol))] (PG) (Avanti Polar Lipids) and 1,1′,2,2′-tetratetradecanoyl cardiolipin (CL) (Avanti Polar Lipids). Solvents were evaporated for 15 min by rotary evaporation. The preparation was resuspended in 20 mM Tris pH 7.5 and then sonicated (Branson B200, Thermo Fisher Scientific, Stockholm, Sweden) in a cold-water bath, twice during 2 min and once during 5 min, to form mixed micelles. The standard reaction for the activity assay contained 20 mM CHAPS, 10 mM PG, 1 mM CL, 0.5 µg·mL^−1^ LPS^TRUNC^, 25 mM MgCl_2_, 6 µM UDP-Glc* (PerkinElmer, Stockholm, Sweden) and His-WaaG in a total volume of 40 µL. The reaction was initiated by adding purified His-WaaG (0.1 mg·mL^−1^) and was incubated for different time points at room temperature. The compounds L1 to L3 were solubilized in DMSO (1 M stock solution) and then added to the reactions to a final concentration of 25 mM. The reaction was stopped by adding 2× Laemmli buffer (Life Technologies, Stockholm, Sweden). An aliquot of 10 µL was loaded per lane and separated by SDS-PAGE on a Novex 4%–12% Bis-Tris gel (Life Technologies). The gels were dried for 60 min at 80 °C (SGD 2000, Savant), and the efficiency of Glc* transfer from UDP-Glc* to LPS^TRUNC^ was determined by autoradiography (Fujifilm FLA-3000) and densitometric analysis using ImageJ software (Fujifilm, Stockholm, Sweden). To determine the specific activity of WaaG, we first calculated the ratio of product formed using the values from the densitometric analysis and the equation (LPS^TRUNC^-Glc*/(UDP-Glc* + LPS^TRUNC^-Glc*)). We then multiplied this ratio by the concentration of UDP-Glc* added to the reaction and normalized the values by the reaction time and the amount of protein added. To calculate the IC_50_ of L1, we titrated it in at different concentrations and then calculated the specific activity relative to the DMSO control. These values were plotted, and a dose-response curve was fitted using GraphPad v5. The IC_50_ was calculated as the midpoint between the DMSO control (which was assumed to be 100% activity) and the activity at 25 mM of L1 (which was assumed to be 0% activity).

## 4. Conclusions

In this study, we have established an activity assay for monitoring the catalytic activity of WaaG from *E. coli*. The assay uses SDS-PAGE and autoradiography to monitor the transfer of a ^14^C-labeled glucose from UDP-Glc* to partially-synthesized LPS (purified from a ∆*waaG* strain of *E. coli*). In optimizing the assay, we noted that the addition of certain lipids, such as PG and CL, as well as the detergent CHAPS significantly increased WaaG activity. Most significantly, we used the assay to show that 4-(2-amino-1,3-thiazol-4-yl)phenol inhibits WaaG activity (IC_50_ 1.0 mM). Since L1 is a non-substrate-like inhibitor and because it is a small molecule (192 Da), it can potentially be further developed into an inhibitor of WaaG [28,29]. One consideration is that L1 competes with the uridine-binding site (or donor site) of WaaG and that a number of human GT-B enzymes also have uridine-binding sites. Thus, it would need to be chemically expanded towards the LPS binding site (or acceptor site) of WaaG. It is worth noting that 2-aminothiazole-based scaffolds, like L1, are often identified in binding screens and have been developed into marketed drugs, such as antibiotics, dopamine agonists, β_3_ adrenoreceptor agonists and drugs to treat myeloid leukemia [30]. Thus, this study represents a significant step on the path towards an inhibitor of WaaG by fragment-based lead discovery.

## Abbreviations

The following abbreviations are used in this manuscript

CHAPS: 3-[(3-cholamidopropyl)dimethylammonio]-1-propanesulfonate
CL: cardiolipin
DHPC: 1,2-dihexanoyl-*sn*-glycero-3-phosphocholine
Hep: l-*glycero*-d-*manno*-heptose
HepII: l-*glycero*-d-*manno*-heptose-II
Kdo: 3-deoxy-d-*manno*-oct-2-ulosonic acid
LPS: lipopolysaccharide
PA: phosphatidic acid
PC: phosphatidylcholine
PE: phosphatidylethanolamine
PG: phosphatidylglycerol
PS: phosphatidylserine
